# Transoral videolaryngoscopic surgery for hypopharyngeal anaplastic metastasis from papillary thyroid carcinoma

**DOI:** 10.1210/jcemcr/luag003

**Published:** 2026-02-19

**Authors:** Yuya Yokoyama, Naoki Nishio, Mai Sugiura, Katsunao Suzuki, Akihisa Wada, Michihiko Sone

**Affiliations:** Department of Otorhinolaryngology, Nagoya University Graduate School of Medicine, Nagoya 466-8550, Japan; Department of Otorhinolaryngology, Nagoya University Graduate School of Medicine, Nagoya 466-8550, Japan; Department of Otorhinolaryngology, Nagoya University Graduate School of Medicine, Nagoya 466-8550, Japan; Department of Otorhinolaryngology, Nagoya University Graduate School of Medicine, Nagoya 466-8550, Japan; Department of Otorhinolaryngology, Nagoya University Graduate School of Medicine, Nagoya 466-8550, Japan; Department of Otorhinolaryngology, Nagoya University Graduate School of Medicine, Nagoya 466-8550, Japan

**Keywords:** papillary thyroid carcinoma, anaplastic thyroid carcinoma, hypopharyngeal metastasis, transoral videolaryngoscopic surgery, BRAF/MEK inhibitors

## Abstract

We report an extremely rare case of hypopharyngeal metastasis with anaplastic transformation (anaplastic thyroid carcinoma) originating from papillary thyroid carcinoma, demonstrating successful tumor control using minimally invasive transoral surgery combined with targeted therapy. A 78-year-old man with a history of papillary thyroid carcinoma treated with total thyroidectomy and radioactive iodine 10 years earlier presented with sore throat and difficulty swallowing. Fiberoptic examination revealed a 25-mm mass on the posterior hypopharyngeal wall causing airway compromise, necessitating an emergency tracheotomy. Biopsy confirmed hypopharyngeal metastasis with anaplastic thyroid carcinoma. Given his comorbidities (chronic leukemia, severe pulmonary arterial hypertension), he underwent transoral videolaryngoscopic surgery to achieve local tumor control while preserving swallowing function. Postoperative genetic testing detected a *BRAF* V600E pathogenic variant, leading to treatment with encorafenib and binimetinib, initiated on postoperative day 24. Six months after surgery and targeted therapy, fiberoptic examination and contrast-enhanced computed tomography showed no evidence of recurrent disease; the tracheostomy was closed, and the patient maintained normal swallowing and quality of life. This case illustrates that a surgery-first, function-preserving approach integrated with early molecular-targeted BRAF/MEK inhibition can achieve durable local control and maintain laryngopharyngeal function, even when anaplastic transformation occurs within a hypopharyngeal metastatic focus.

## Introduction

Papillary thyroid carcinoma (PTC) is known to progress slowly and generally has a relatively favorable prognosis. However, PTC can undergo anaplastic transformation (anaplastic thyroid carcinoma [ATC]) during long-term follow-up, which is considered one of the most aggressive malignant behaviors among solid tumors. ATC is associated with an extremely poor prognosis, with survival typically ranging from 6 to 12 months, even with multimodal treatments such as surgery, external beam radiation therapy, or targeted drug therapy [1]. Moreover, rapid tumor progression in the head and neck region often causes severe dysfunction, including bleeding, airway obstruction, and swallowing difficulties, significantly reducing quality of life. Therefore, both effective carcinoma treatment and adequate functional management are essential in patients with ATC.

PTC often metastasizes to cervical lymph nodes, lungs, and bones, whereas metastasis to the pharynx or larynx is extremely rare. Only a few cases of pharyngeal or laryngeal metastasis of PTC have been reported [2-6], and documentation of hypopharyngeal metastasis with ATC remains extremely limited. Managing ATC in unusual anatomical locations poses unique challenges, especially when aggressive surgery is contraindicated because of comorbidities. Integrating minimally invasive techniques with targeted therapies may represent a useful option in personalized care for selected patients. Here, we present a case of hypopharyngeal metastasis with anaplastic transformation that occurred during the follow-up of PTC. The patient achieved favorable tumor control through transoral videolaryngoscopic surgery (TOVS) combined with BRAF/MEK inhibitor therapy.

## Case presentation

A 78-year-old man presented with a sensation of throat obstruction and dyspnea. His medical history included total thyroidectomy and radioactive iodine therapy for PTC performed 10 years earlier, chronic leukemia, drug-induced pulmonary arterial hypertension, cerebral infarction, and angina pectoris. Five years earlier, he underwent right lateral neck dissection for nodal recurrence. Four years earlier, when mediastinal lymph node and pulmonary metastases were detected, a biopsy of a concurrent erythematous lesion in the right hypopharynx revealed granulation tissue with no evidence of malignancy. These metastases grew slowly and were managed with careful observation during treatment for chronic leukemia.

**Figure 1:**
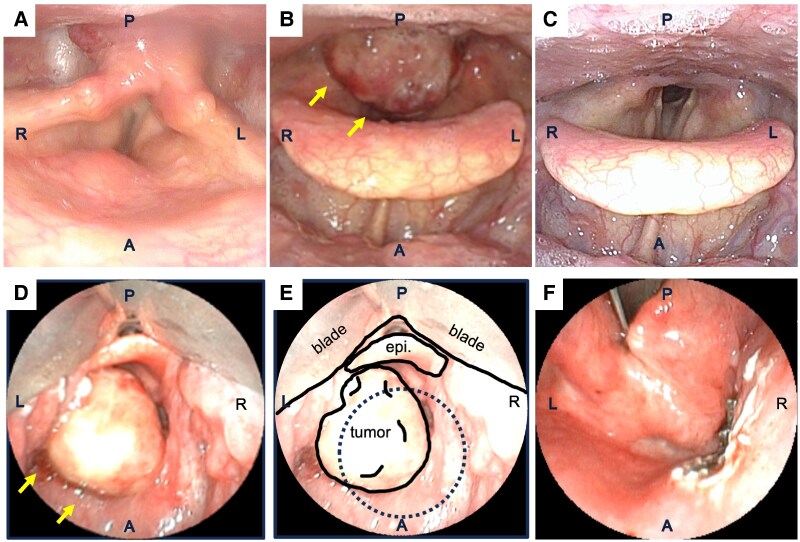
Laryngoscopic and intraoperative photographs. A, anterior region; P, posterior region; L, left side; R, right side; epi, epiglottis. Upper panels (A-C) show sequential laryngoscopic findings, and lower panels (D-F) show intraoperative findings during transoral videolaryngoscopic surgery (TOVS). (A) Laryngoscopic image 3 years before presentation. (B) Laryngoscopic image at current presentation. (C) Laryngoscopic image 6 months after treatment. (D, E) Intraoperative photographs during TOVS before resection. (D) Overview of the tumor location; (E) schematic illustration showing the surgical approach and anatomical orientation during TOVS. The blade position and epiglottis (epi) are indicated. (F) Postresection view corresponding to the dotted area in panel E, showing the resected tumor bed.

In the current year, a 25-mm lesion appeared on the posterior wall of the hypopharynx, causing the patient to experience throat obstruction and prompting referral. Flexible laryngoscopy revealed a midline posterior hypopharyngeal mass protruding into the laryngeal inlet, resulting in airway narrowing (Figure 1). Palpation identified a 20-mm subcutaneous mass with limited mobility, located slightly to the right of the midline in the anterior neck.

## Diagnostic assessment

**Figure 2:**
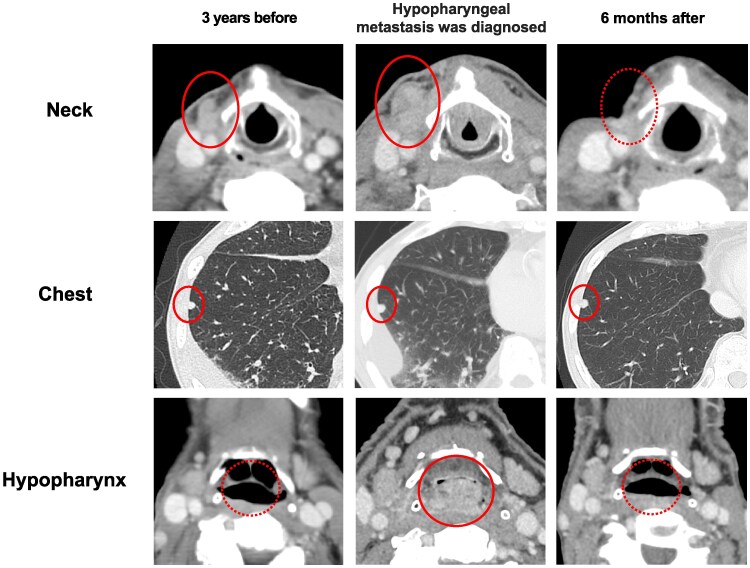
Serial CT imaging from 3 years before treatment to 6 months after treatment. Serial computed tomography (CT) images showing disease progression and treatment response. Upper row: Contrast-enhanced cervical CT images at 3 years before, at current presentation, and 6 months after, showing changes in the anterior cervical lymph node metastasis. Middle row: Chest CT images at 3 years before, at current presentation, and 6 months after, showing lung metastasis. Lower row: Contrast-enhanced hypopharyngeal CT images at 3 years before, at current presentation, and 6 months after, showing changes in the hypopharynx metastasis.

Laboratory tests showed white blood cell count 7.9 × 10³/μL (SI: 7.9 × 10⁹/L) (reference range, 3.3-8.6 × 10³/μL [SI: 3.3-8.6 × 10⁹/L]), TSH 5.81 μIU/mL (SI: 5.81 mIU/L) (reference range, 0.35-4.94 μIU/mL [SI: 0.35-4.94 mIU/L]), free T3 2.24 pg/mL (SI: 3.44 pmol/L) (reference range, 1.71-3.71 pg/mL [SI: 2.63-5.70 pmol/L]), and free T4 1.20 ng/dL (SI: 15.4 pmol/L) (reference range, 0.70-1.48 ng/dL [SI: 9.0-19.1 pmol/L]). Contrast-enhanced computed tomography (CT) demonstrated a 25-mm, well-demarcated, high-attenuation tumor on the posterior hypopharyngeal wall causing significant airway compromise. Margins were smooth with no invasion of prevertebral musculature. The mediastinal lymph node swelling and pulmonary nodules remained stable compared to CT images obtained 3 years before. Additionally, a separate, ill-defined, 20-mm enhancing lesion was noted in the right anterior neck (Figure 2).

Due to progressive airway obstruction, an emergency tracheotomy and tumor biopsy were performed under local anesthesia. Subsequently, the biopsy confirmed hypopharyngeal metastasis with ATC. The initial specimen was insufficient for OncoMine *BRAF* mutation analysis.

## Treatment

Given the rapid hypopharyngeal tumor growth, the presence of multiple pulmonary metastases and severe comorbidities, and the high risk of lenvatinib because of the patient's severe pulmonary arterial hypertension and potential cardiovascular adverse effects, we elected to perform TOVS. Using a mouth-opening device and 3D Tip Curved Videoscope (ENDOEYE FLEX, Olympus, Japan), the pharyngeal tumor was clearly identified under general anesthesia. The hypopharyngeal tumor measured 25 mm at the posterior wall of the hypopharynx. After mucosal incision with an electrosurgical knife, the tumor was successfully removed without any complications. Subsequently, the right anterior neck tumor was also removed (Figure 1).

**Figure 3:**
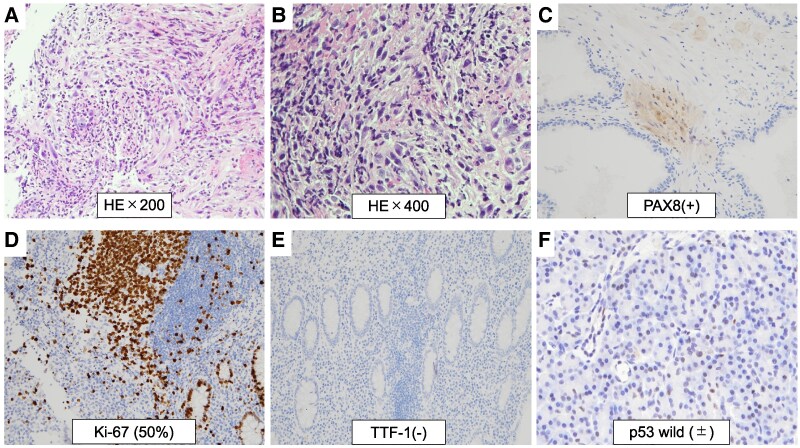
Histopathological findings of hypopharyngeal lesion confirming ATC. Histopathological examination of the hypopharyngeal surgical specimen. HE staining: (A) Low-power magnification (×200) showing diffuse infiltration of atypical cells. (B) High-power magnification (×400) showing tumor cells with prominent nuclear enlargement and atypical spindle cells. Immunohistochemical staining: (C) PAX8-positive nuclear staining (×200), (D) Ki-67 proliferation index of approximately 50% (×200), (E) TTF-1-negative staining (×200), and (F) p53 wild-type pattern (±) (×400).

Histopathological examination of the hypopharyngeal specimen revealed densely packed atypical spindle cells arranged in fascicles with slit-like spaces. The anaplastic transformation was observed throughout the entire resected tumor specimen. Immunohistochemical analysis demonstrated PAX8 positivity, focal thyroglobulin positivity, AE1/AE3 negativity, a Ki-67 index of 50%, TTF-1 negativity, and p53 wild-type pattern, confirming hypopharyngeal metastasis with ATC (Figure 3). The resection margins were positive for anaplastic tumor cells.

The anterior neck tumor exhibited papillary structures composed of atypical epithelial cells with nuclear grooves and inclusions. No anaplastic transformation was observed, and the areas showing squamous differentiation tested positive for PAX8, TTF-1, and p40. OncoMine *BRAF* mutation analysis of the resected tumor detected a *BRAF* V600E mutation.

## Outcome and follow-up

The postoperative course proceeded without complications, and oral intake was resumed on postoperative day 2. Given the presence of the *BRAF* V600E pathogenic variant, combined therapy with encorafenib (450 mg daily) and binimetinib (90 mg daily) was initiated on postoperative day 24. Six months after the initiation of targeted therapy, endoscopic and imaging assessments revealed no evidence of recurrence; airway patency was preserved, and the tracheostomy site remained closed. The patient continues to receive outpatient therapy with good tolerance.

## Discussion

We report an extremely rare case of hypopharyngeal metastasis with ATC originating from PTC, successfully managed with TOVS and targeted therapy. Currently, only a few cases of hypopharyngeal metastasis from PTC have been reported, and optimal management strategies for such metastases remain unclear [2-6]. TOVS is a minimally invasive technique developed in Japan for T1-T3 oropharyngeal and hypopharyngeal carcinoma using curved laryngoscopy for wide exposure and transoral en bloc resection. Because of the rapid growth of hypopharyngeal metastasis with ATC, the patient showed swallowing dysfunction and suffered from airway obstruction. Moreover, the patient had severe pulmonary hypertension and declined extensive tumor resection, such as total laryngopharyngectomy. Therefore, we selected a minimally invasive resection followed by targeted therapy. Although adequate safety margins were not obtained, the tumor was removed without complications, resulting in good oral intake and favorable quality of life outcomes. Multimodal treatment, including surgery, external beam radiation therapy, and targeted drug therapy, is essential for managing hypopharyngeal metastasis with ATC.

Regarding systemic therapy, molecular targeted therapies are approved for PTC and ATC, with BRAF/MEK inhibitor combinations effective for *BRAF* V600E-positive ATC (30%-40% of cases). For improved survival outcomes, a combination of surgery and BRAF/MEK inhibitors has been reported in patients with ATC [7, 8]. One study from the United States indicated that, in patients with *BRAF* V600E mutation-positive ATC, treatment with dabrafenib and trametinib (± immune checkpoint inhibitor therapy) followed by curative resection resulted in a 1-year overall survival rate of 94% and a 1-year progression-free survival rate of 84%. This combined approach allowed surgeons to reduce resection extent and contributed to improved survival outcomes [8]. Because of an insufficient initial biopsy specimen, gene pathogenic variant analysis could not be performed. After surgical resection for hypopharyngeal metastasis with ATC using the transoral technique, *BRAF* pathogenic variant was detected in the final pathological results. At 6 months after treatment with BRAF/MEK inhibition, the patient had no airway obstruction or hoarseness, and no recurrence was observed. The decision to pursue a surgery-first approach rather than neoadjuvant BRAF/MEK inhibition was based on several factors: the acute airway compromise requiring immediate intervention, the need to rapidly debulk the hypopharyngeal mass to secure swallowing and airway function, and the need to obtain adequate tissue for comprehensive pathological and molecular assessment.

Regarding the mechanism of hypopharyngeal metastasis, the common pattern of metastasis in thyroid carcinoma is lymphatic spread, observed in 30% to 80% of cases. Distant metastasis often occurs mainly in the lung, bone, skin, or brain. When lesions involve the recurrent laryngeal nerve, larynx, pharynx, trachea, or esophagus, it is considered direct tumor invasion [9]. The possible mechanisms for hypopharyngeal metastasis in this case are as follows: (1) hematogenous metastasis—carcinoma cells enter the systemic circulation via the venous system and reach the hypopharyngeal tissue through the rich vascular plexus of the pharynx and (2) retrograde lymphatic metastasis—due to abnormalities in cervical lymphatic flow, carcinoma cells may spread retrogradely through the lymphatic vessels. In this case, since the hypopharyngeal metastasis appeared 10 years after the initial surgery, and mediastinal lymph node and lung metastases were also observed, hematogenous metastasis is considered the most likely mechanism. The hypopharynx is a site with abundant blood flow, and it is presumed that carcinoma cells that reached it hematogenously implanted in the submucosal tissue. Regarding hypopharyngeal metastasis of thyroid carcinoma, a few cases of metastasis from papillary thyroid carcinoma have been reported; however, reports of metastases involving anaplastic carcinoma to the hypopharynx are extremely rare [2, 3, 5, 6] (Table 1). Considering the patient's overall condition, a minimally invasive local excision via TOVS was selected, and favorable tumor control has been achieved postoperatively with molecular targeted therapy. This case suggests that even in the extremely rare scenario of hypopharyngeal metastasis from anaplastic thyroid carcinoma, appropriate treatment strategies can achieve tumor control while preserving the patient's quality of life. In such situations, TOVS can be considered as a minimally invasive option for selected patients with hypopharyngeal metastases from thyroid carcinoma, particularly when combined with appropriate molecular targeted therapy. This approach may be especially relevant for medically fragile patients in whom traditional extensive surgical procedures pose prohibitive risks. However, the follow-up period of 6 months in this case is limited, and longer term observation is necessary to fully assess the durability of disease control and survival outcomes.

**Table 1:** Comparison of reported cases of hypopharyngeal metastasis from thyroid carcinoma

Ref	Age/sex	Site(s)	Pathology	Treatment	Outcome
[2]	41/M	Posterior pharyngeal wall, aryepiglottic fold	PTC	Total thyroidectomy + bilateral neck dissection/hypopharyngeal lesion: not treated	Debulked; lost to follow-up
[3]	50/M	Right aryepiglottic fold and right lateral wall of the pyriform sinus	PTC	Microlaryngeal/CO2 laser resection + total thyroidectomy + modified neck dissection + I-131	resected; regular follow-up
[4]	70/F	Lateral hypopharyngeal wall + parapharyngeal space	PTC	Cervical approach; partial hypopharyngeal mucosal resection; primary closure of the mucosal defect	resected; ND
[5]	58/M	Posterior hypopharyngeal wall	PTC	Surgery (ND)	ND
[6]	70/M	Posterior hypopharyngeal wall	PTC	ND	ND
NA	78/M (present case)	Posterior hypopharyngeal wall	ATC	Transoral videolaryngoscopic surgery followed by BRAF/MEK inhibitors	No recurrence at 6 months

Abbreviations: ATC, anaplastic thyroid carcinoma; I-131, radioactive iodine-131; NA, not available; ND, no data; PTC, papillary thyroid carcinoma; Ref, reference.

## Learning points

- Hypopharyngeal metastasis with anaplastic transformation from PTC, although extremely rare, can present as a life-threatening emergency requiring immediate multidisciplinary management.
- TOVS represents a viable minimally invasive option for hypopharyngeal metastases in high-risk patients, enabling rapid local control while preserving critical swallowing and voice functions.
- Early molecular profiling and prompt initiation of targeted BRAF/MEK inhibition are crucial for optimizing outcomes in *BRAF* V600E-positive ATC, even in unusual metastatic locations.
- Multidisciplinary treatment planning integrating function-preserving surgery with targeted systemic therapy can achieve excellent disease control while maintaining quality of life in complex clinical scenarios.

## Contributors

Y.Y. conceived and designed the project. Y.Y., N.N., and K.S. were involved in the diagnosis and management of this patient. Y.Y., N.N., and K.S. were responsible for data curation. Y.Y. and N.N. drafted the initial version of the manuscript. All authors (Y.Y., N.N., M.S., K.S., A.W., M.S.) developed research plans and participated in research design, manuscript development, editing, and completion of the manuscript. All authors (Y.Y., N.N., M.S., K.S., A.W., M.S.) were responsible for manuscript revision and approved the submitted version.

## Data Availability

Original data generated and analyzed for this case report are included in this published article.
